# Pulsed Field Ablation of Atrial Fibrillation: A Comprehensive Review

**DOI:** 10.31083/j.rcm2411337

**Published:** 2023-11-30

**Authors:** Carlos D. Matos, Carolina Hoyos, Andres F. Miranda-Arboleda, Juan C. Diaz, Daniela Hincapie, Carlos Patino, Ricardo H. Hernadez, Paul C. Zei, Jorge E. Romero, Jose Osorio

**Affiliations:** ^1^Cardiac Arrhythmia Service, Division of Cardiovascular Medicine, Brigham and Women’s Hospital & Harvard Medical School, Boston, MA 02115, USA; ^2^Cardiac Arrhythmia Center, Division of Cardiology, Las Vegas, Medellin 050021, Colombia; ^3^HCA Electrophysiology, Mercy Hospital, Miami, FL 33133, USA

**Keywords:** atrial fibrillation, catheter ablation, pulsed-field ablation

## Abstract

Pulsed-field ablation (PFA) has emerged as a promising nonthermal ablation alternative for treating atrial fibrillation (AF). By delivering ultra-rapid high-energy electrical pulses, PFA induces irreversible electroporation, selectively targeting myocardial tissue while sparing adjacent structures from thermal or other damage. This article provides a comprehensive review of multiple pre-clinical studies, clinical studies, and clinical trials evaluating the safety, efficacy, and long-term outcomes of PFA in various settings and patient populations. Overall, the reviewed evidence highlights PFA’s potential as a revolutionary ablation strategy for AF treatment. Offering comparable procedural efficacy to conventional ablation methods, PFA distinguishes itself with shorter procedure times and reduced risks of complications such as phrenic nerve palsy and potential esophageal injury. While further research is warranted to establish long-term efficacy, PFA’s distinct advantages and evolving clinical evidence suggest a promising future for this novel nonthermal ablation approach. As PFA continues to advance, it has the potential to transform AF ablation procedures, providing a safer alternative for patients with atrial fibrillation.

## 1. Introduction 

Atrial fibrillation (AF) is one of the most common cardiovascular diagnoses and a common cardiovascular comorbidity, especially in elderly populations. The current worldwide prevalence of AF is estimated to be more than 37 million, with an incidence of over 2.8 million cases per year [1]. Stroke risk prevention strategies are one of the mainstays in the management of AF, which includes risk stratification, oral anticoagulation, and left atrial appendage occlusion procedures [2]. Another key element in the treatment of AF is rate and rhythm control, as symptomatic AF has been shown to lower the quality of life and increase the risk of hospitalizations [3]. Furthermore, uncontrolled AF often leads to AF-induced cardiomyopathy [4, 5, 6]. In this context, catheter ablation (CA) has evolved to become a suitable first-line management strategy for AF [7, 8].

Catheter ablation of AF is mostly performed using radiofrequency (RF), or less commonly cryotherapy, as an energy source [9]. RF ablation (RFA) relies on resistive tissue heating to generate a controlled scar, leading to the electrical isolation of the desired structure [10], which usually comprises the pulmonary veins and sometimes the posterior wall of the left atrium or other structures [11]. RFA has been proven superior to AADs in several randomized control trials (RCT) [12, 13, 14]. Cryoablation is another frequently used CA technique for the treatment of AF, which relies on extreme tissue cooling (typically via a cryoballoon) to isolate the desired structure with similar targets to RFA [15]. The latter has also been proven superior to AAD in multiple RCTs [16, 17, 18], with an efficacy and safety profile similar to RFA [19].

**Fig. 1.**
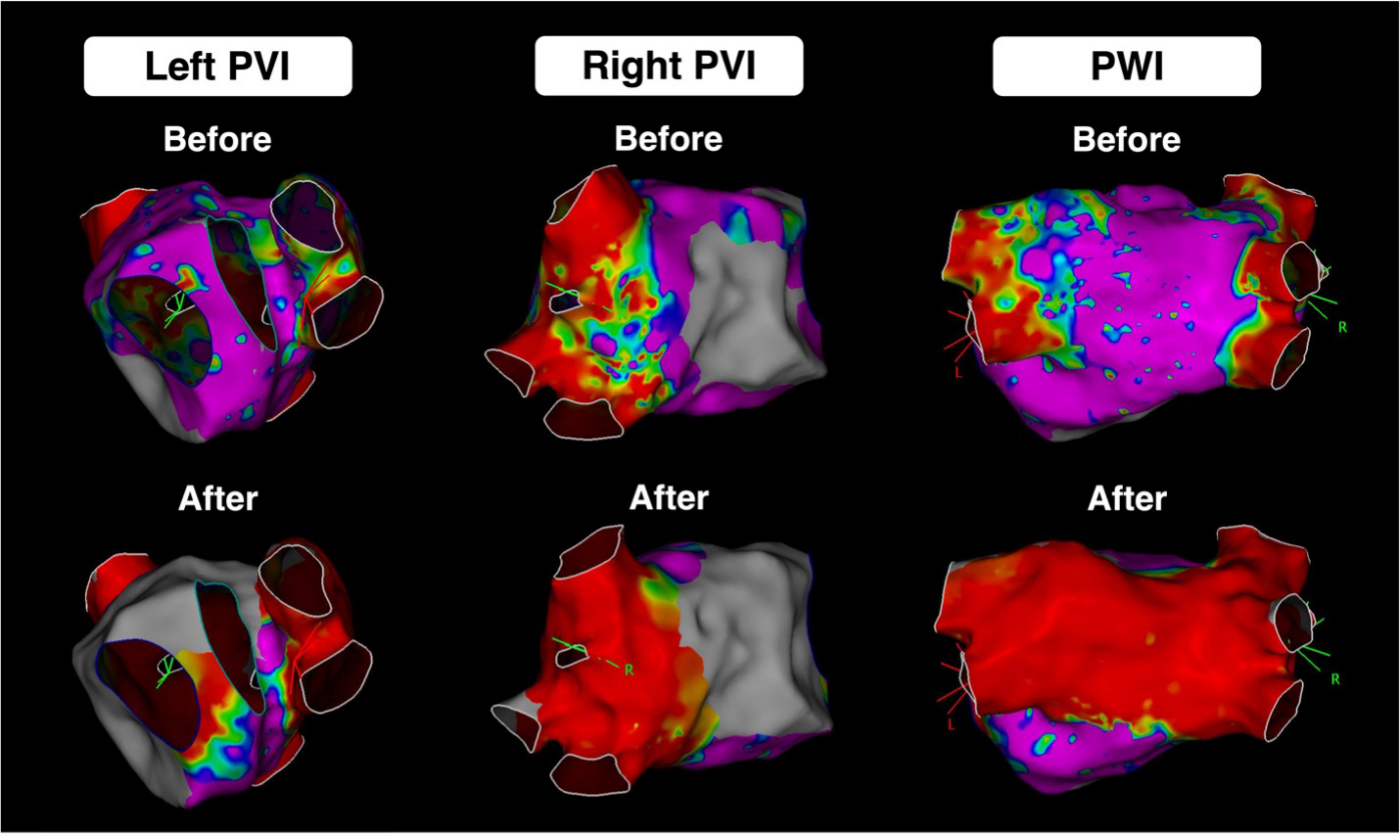
**Pulmonary vein and posterior wall isolation performed with PFA. **Electroanatomic mapping of the left- and right-sided pulmonary veins as well as the posterior wall of the left atrium before and 3 months after pulmonary vein and posterior wall isolation with the Farapulse PFA System. Courtesy of Dr. Jorge E Romero. PFA, pulsed-field ablation; PVI, pulmonary vein isolation; PWI, posterior wall isolation.

While CA has been proven beneficial, the aforementioned ablation strategies have been associated with several complication risks. These include pericardial tamponade/effusion, pulmonary vein stenosis, atrioesophageal fistula, and reversible vs. permanent phrenic nerve injury [20]. As the use of CA as a first-line therapy for AF gained popularity, the importance of minimizing potential procedural complications increased. In consequence, pulsed-field ablation (PFA) has been recently proposed as an alternative with the potential to reduce or eliminate several severe complications associated with CA of AF (Fig. 1) [21].

## 2. The Origin of Pulsed Field Ablation

In the early days of cardiac ablation, cellular death was induced by using diagnostic catheters to deliver direct current (DC) to the desired tissue. This strategy was initially used to ablate accessory pathways and atrioventricular nodal reentrant tachycardias [22, 23, 24]. At that time, very high levels of energy were delivered via a monophasic unipolar defibrillation wave to achieve effective tissue modification. This high-level energy often resulted in major adverse outcomes, including myocardial perforation and tamponade or the formation of heterogeneous proarrhythmic scars [25, 26]. Subsequent studies, however, demonstrated that by using lower energy levels, successful ablation could be achieved while decreasing the risk of adverse events [27, 28, 29, 30]. However, simultaneous to the evolution of settings optimization of DC ablation, RFA emerged as an alternative ablation technique capable of a higher control of the total energy delivered to the tissue with significantly decreased complication rates [31, 32].

Since then, RFA has evolved by using objective lesion assessment [33], optimizing catheter stability via ventilation and pacing strategies [33, 34], and optimizing power delivery [35, 36]. Nonetheless, RFA is not without its drawbacks, as it induces non-selective thermal damage, which could result in significant harm to the additional nearby tissues beyond the myocardium. Regarding the ablation of AF, this could lead to complications such as pulmonary vein stenosis, esophageal ulceration, atrioesophageal fistulas, and injury to adjacent nerves (like the phrenic nerve) or coronary vessels [20]. Moreover, by relying on thermal energy delivery for lesion formation, it is substantially influenced by blood flow surrounding the catheter due to its cooling effect, which often has to be overcome by increasing power and contact force, thus increasing the risk of steam pops and heart perforation [37, 38]. Cryoablation later emerged as an alternative energy source for CA of AF. Notwithstanding this being a different approach, it is associated with comparable efficacy and safety to RFA [19], but the use of the balloon configuration is primarily limited to pulmonary vein isolation (PVI). More recently, with the aim of improving outcomes regarding complications, attention has been brought back to DC as a source of energy for CA. PFA involves using short pulses of high energy to create electric fields, which can theoretically target the myocardium without damaging nearby structures.

## 3. Mechanism of Action of PFA

**Fig. 2.**
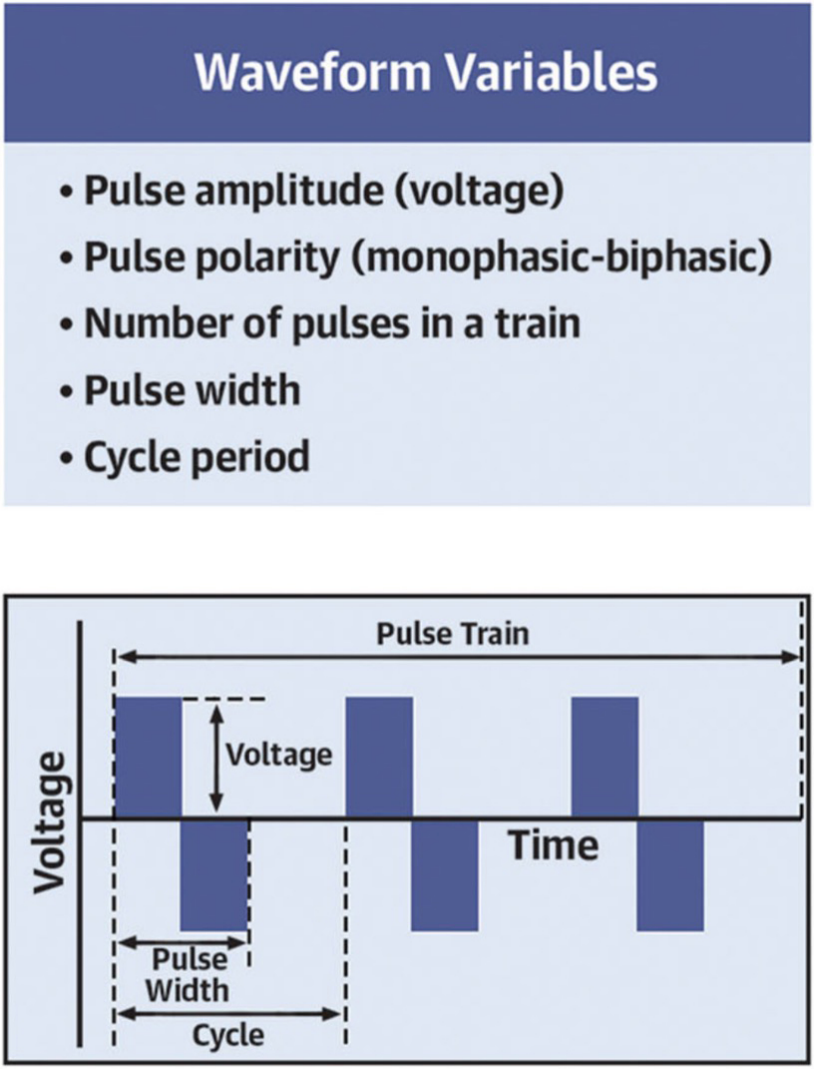
**Pulsed-field ablation waveform parameters**. Waveform modifiable parameters determine the target tissue, as well as the lesion’s durability and extension. Reprinted from JACC: EP, 33(7), Romero *et al*., Pulsed-field ablation: What are the unknowns and when will they cease to concern us?, with permission from Elsevier [21].

**Fig. 3.**
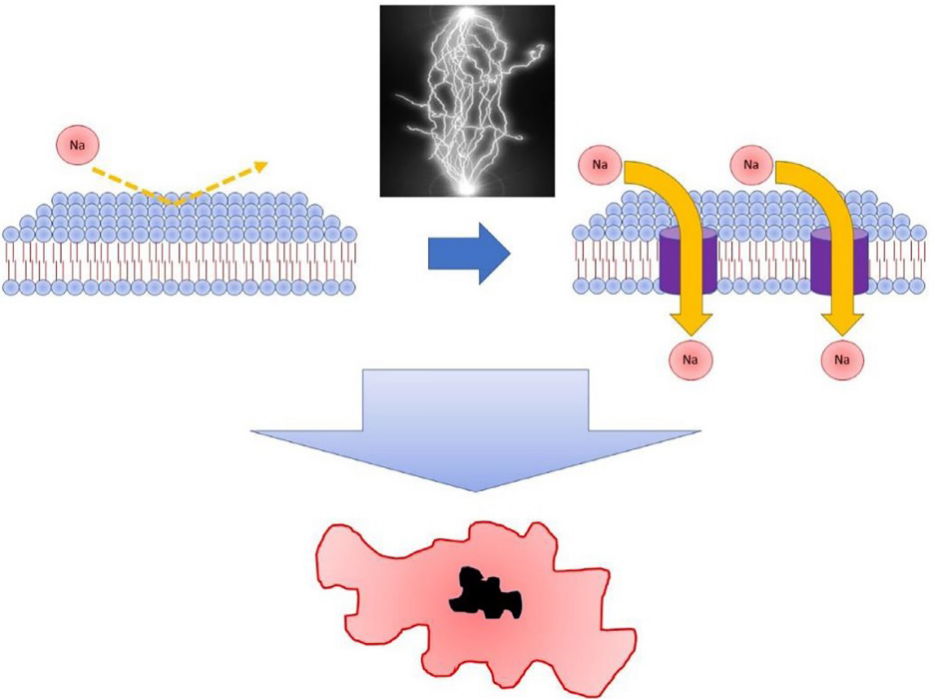
**Irreversible electroporation (IRE)**. Illustration of the IRE process, as part of which exposure to a predetermined energy field results in increased cell membrane permeability leading to cardiac death. Reprinted from JACC: EP, 32(6), Romero *et al*., Pulsed field catheter ablation in atrial fibrillation, with permission from Elsevier [41].

PFA relies on electroporation, rather than thermal energy delivery, to create effective isolation lesions. It mainly consists of a process where the cell’s membrane permeability is increased by subjecting it to an electric field with predetermined characteristics and delivery patterns [39]. Cellular membranes are composed of a resilient phospholipid bilayer that impedes the diffusion of polar molecules, thus protecting the cell and supporting its essential functions. However, when subjected to external electric fields, said protective layer is compromised, resulting in the creation of nanopores. These nanopores result in the diffusion of ionic particles through the cellular membrane [40]. The degree and duration of this increased cellular membrane permeability depend on the amplitude, width, number of pulses, waveform type (biphasic or monophasic), and pulse cycle length of the delivered PFA (Fig. 2, Ref. [21]). When the degree and duration of this cellular membrane modification are optimized, cellular death can be achieved, a process named irreversible electroporation (IRE) (Fig. 3, Ref. [41]). The field parameters capable of IRE are referred to as the IRE threshold [42]. The occurrence of cellular death due to field exposures surpassing the IRE threshold can be attributed to mechanisms such as adenosine triphosphate (ATP) depletion, impairment of ion channels, increased calcium influx, and the alteration of cellular homeostasis [43, 44, 45].

**Fig. 4.**
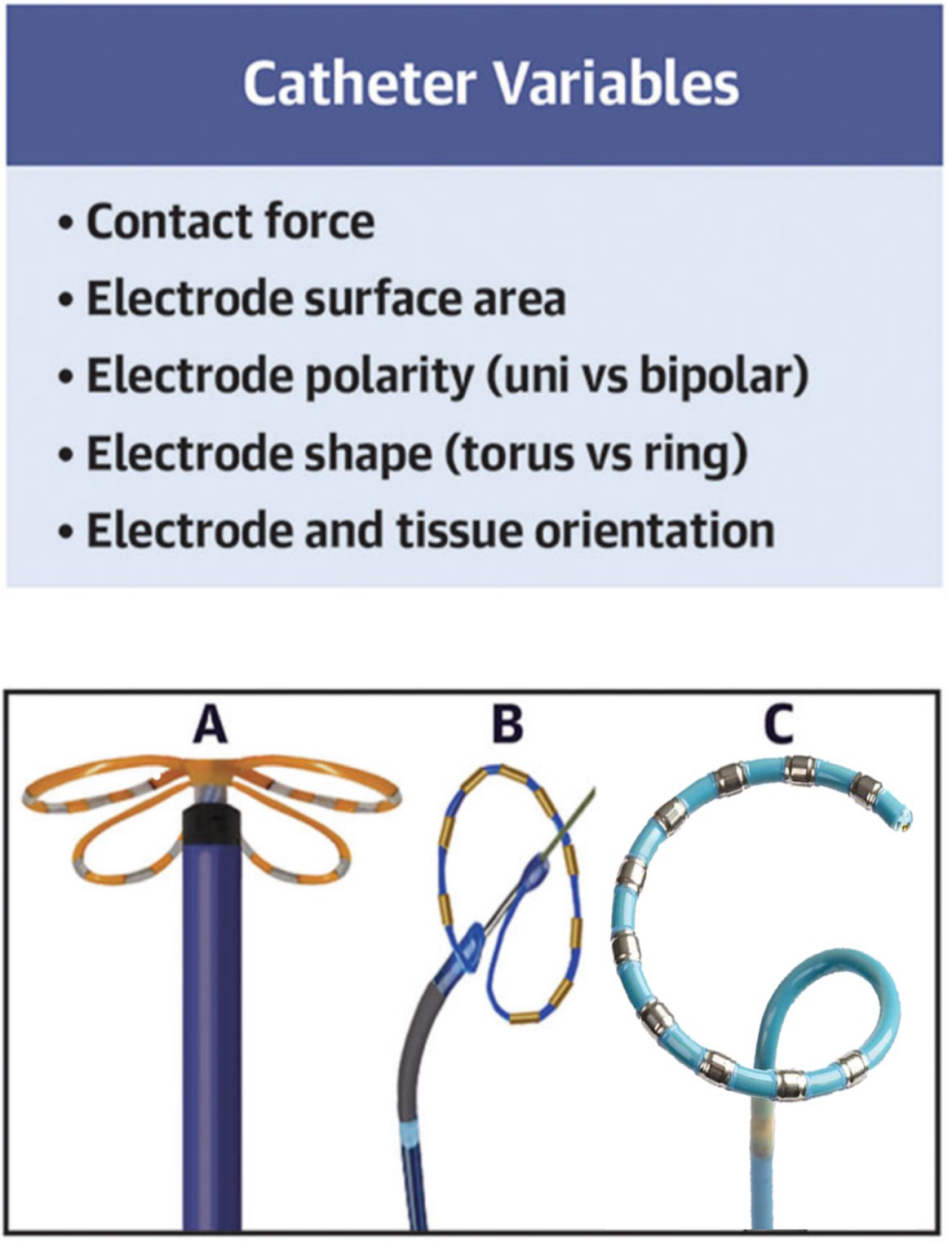
**Pulsed-field ablation (PFA) catheter parameters**. Catheter shape, electrode disposition, and mechanism of deployment and navigation determine the target tissue, as well as the lesion’s durability and extension. (A) PFA Farawave catheter (Farapulse, Boston Scientific). (B) PFA, Pulmonary vein Ablation Catheter GOLD (PVAC GOLD; Medtronic, Inc.). (C) PFA circular contractable Varipulse ablation catheter (Biosense Webster, Inc.). Reprinted from JACC: EP, 33(7), Romero *et al*., Pulsed-field ablation: What are the unknowns and when will they cease to concern us?, with permission from Elsevier [21].

PFA has emerged as an alternative energy source particularly useful for the CA of AF. It delivers short pulses of high energy that can selectively target tissue without a significant thermal effect. This characteristic is crucial as it avoids denaturation of blood proteins and damage to the extracellular matrix, thus minimizing the possibility of unintended injury to surrounding tissues [46]. To prevent adverse events previously associated with DC ablations, the concept of pulsed biphasic bipolar waveforms was proposed. This strategy resulted in a delivery of energy capable of IRE while confining the affected tissue to the area surrounding the catheter electrode, as well as a significant reduction in general muscle contraction and nerve stimulation [47, 48]. On the other hand, electrode polarity and shape play an important role in lesion formation during PFA. Evidence suggests that unipolar electrode configurations produce deeper lesions than bipolar configurations [49]. Similarly, the shape of the electrode, such as a torus, may enable reduced electric field attenuation and the delivery of deeper lesions compared to standard ring electrodes (Fig. 4, Ref. [21]) [50].

**Fig. 5.**
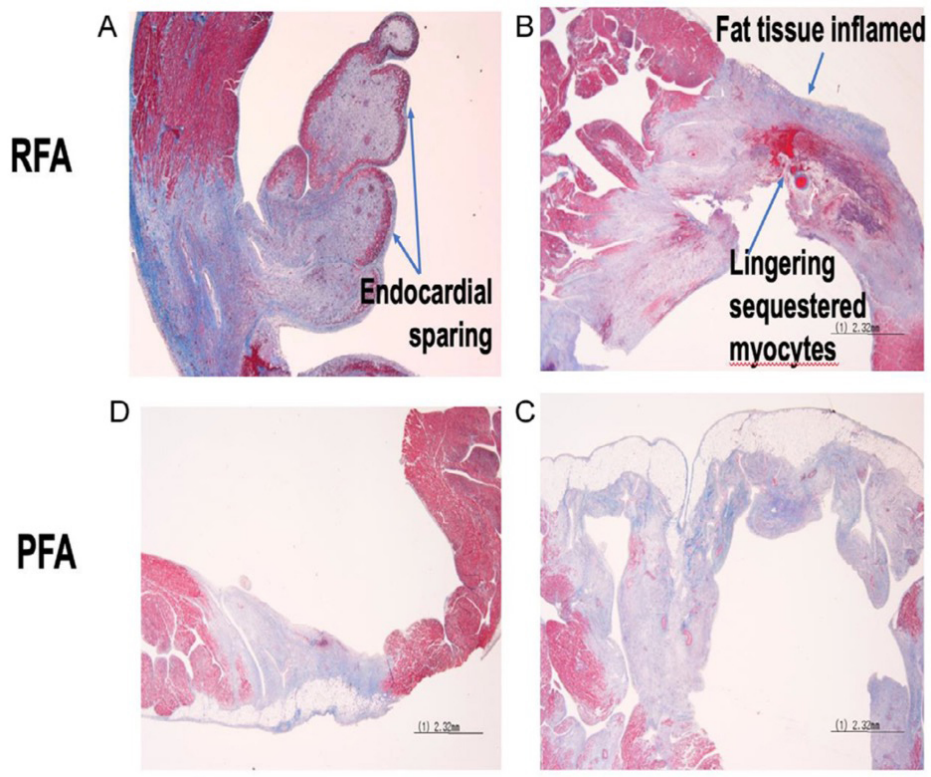
**Radiofrequency ablation (RFA) vs. pulsed-field ablation (PFA) lesion characteristics. **PFA produces more predictable and homogeneous lesions in the myocardium when compared with RFA, particularly on uneven surfaces. (A,B) The growth of radiofrequency (RF) lesions triggers an inflammatory process, which limits its capacity to deliver a successful transmural lesion. (C,D) PFA lesions are consistently more homogeneous than RFA lesions. Reprinted from JACC: EP, 32(6), Romero *et al*., Pulsed field catheter ablation in atrial fibrillation, with permission from Elsevier [41].

Compared to RF energy, PFA has been noted to produce lesions characterized by greater uniformity and homogeneity, especially in irregular substrates, where attaining optional electrode-tissue contact poses a challenge (Fig. 5, Ref. [41]) [51]. Although PFA has demonstrated promising results, further research is warranted to gain a more comprehensive understanding of the impact of different settings on patient safety and effective lesion formation. For instance, synchronizing PFA pulses with the R-wave or cardiac implantable electronic device pacing mitigates the risk of arrhythmia induction [52].

## 4. Potential Benefits of PFA

**Fig. 6.**
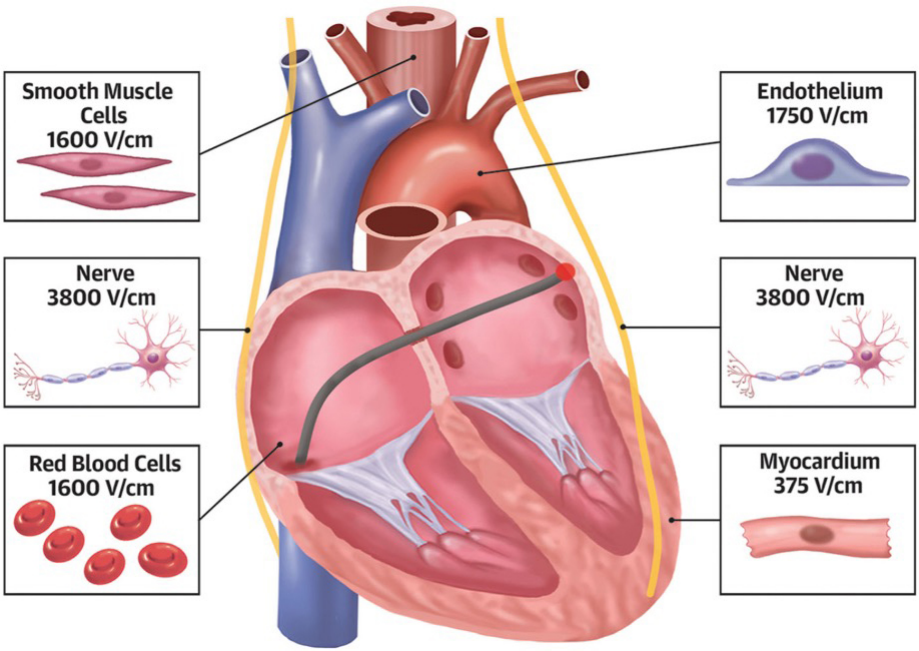
**Pulsed-field ablation (PFA) selectivity**. PFA offers the potential to selectively target myocardial tissue while avoiding detrimental effects on nearby structures such as red blood cells, the phrenic nerve, the esophagus, or coronary vessels. Reprinted from JACC: EP, 33(7), Romero *et al*., Pulsed-field ablation: What are the unknowns and when will they cease to concern us?, with permission from Elsevier [21].

In theory, PFA waveform and catheter characteristics can be modified to selectively target a predetermined tissue, such as the myocardium, while sparing non-target tissues, including the phrenic nerve, esophagus, and blood vessels (Fig. 6, Ref. [21]). Aiming to validate this concept, Hsu *et al*. [50] published results from an *in vivo* study assessing the efficacy and potential safety benefits associated with PFA. They utilized a unique circular irrigated 10-electrode catheter with an integrated generator to deliver PFA in 8 porcine models. The target ablation sites included various locations such as the ostium and inside of the PV, over the phrenic nerve trajectory in the atria, and other areas to check for potential damage. Thirty days after PFA, the models were re-assessed with electroanatomic mapping before a histological evaluation was performed. PFA waveforms were delivered in a bipolar configuration, employing biphasic pulses in trains with an overall application duration of about 250 ms. The electric potential used was 1800V, but the precise allocation of this voltage among multiple electrodes, cycle length, pulse width, and voltage amplitude were not provided [50].

A recently published study demonstrated that PFA spared mediastinal structures immediately and at the 30-day mark despite delivering multiple PFA lesions proximal to them from the endocardial surface. Notably, they observed no instances of PV stenosis, reduction in left ventricular ejection fraction, or injury to the phrenic nerve, mitral valve, or esophagus. The PV ostial lesions were especially effective, with enduring isolation of the PVs observed in all swine. The histological examination revealed circumferential transmural necrosis at these sites [50].

The authors delivered lesions using supratherapeutic parameters from the endocardium proximal to the epicardial location of the phrenic nerve. Remarkably, gross pathology also revealed no signs of injury. This data presents an encouraging prospect, as it opens up the possibility of ablating cardiac tissue at a high risk of collateral tissue damage with RFA or cryoablation (e.g., atrial tachycardias originating from the Crista terminalis, due to its proximity to the phrenic nerve) [50]. Currently, the sole available options for addressing such cases are high-risk procedures that require epicardial access to displace the structure at risk with air, saline solutions, deflectable sheaths, or specialized balloons.

Furthermore, postprocedural assessment with X-ray, intracardiac echocardiography (ICE), and flow velocity revealed no occurrences of PV stenosis, regardless of whether lesions were delivered at the ostium or inside the PVs [50]. This corroborates the findings reported by Howard *et al*. [53], who proposed that PFA could decrease the likelihood of collateral damage compared to RFA. To evaluate PV stenosis more precisely, the authors developed a 3D model using computed tomography angiography, incorporating cross-sectional measurements of the PVs. By measuring PV dimensions before and after the ablation procedure, a comprehensive timeline of the progression of PV stenosis due to RFA was established, while minimal alterations were observed when PFA was used [53]. Various other investigations have assessed the risks associated with PFA around the PVs, with all studies reporting negligible risk of PV stenosis [54, 55, 56, 57].

Moreover, Hsu *et al*. [50] reported that, despite delivering PFA lesions proximal to the esophagus from the aorta, there were no indications of esophageal injury. Upon histological assessment of the esophagus in all subjects following a 30-day post-procedure period, no indications of tissue damage were observed [50]. These findings align with previous *in vivo* investigations, which illustrated the absence of esophageal injury despite the administration of elevated doses of PFA in close proximity to the esophagus [58]. Clinical studies have also supported these results, as they revealed no evidence of unintended injury to the esophagus on post-procedural esophagogastroduodenoscopy (EGD) and chest cardiac magnetic resonance imaging (CMR) [48].

Stewart *et al*. [51] conducted an experimental swine model using a circular catheter (PVAC GOLDTM, Medtronic, Minneapolis, MN, USA) (Fig. 4B) equipped with 9 energy delivery and EGM recording electrodes, which were connected to an experimental PFA generator with the capacity to deliver biphasic pulse trains. PFA involved the delivery of 5 trains consisting of 60 pulses with a potential of 500 V within 10 seconds. The study compared PFA and RFA and found that PFA resulted in a greater reduction of local electrogram amplitude, loss of capture, and transmural lesions without affecting the esophagus or phrenic nerves. Unlike RFA, PFA relies on electrical fields rather than catheter-tissue contact for lesion formation, making tissue contact less crucial for achieving transmural lesions [51].

In a recent study, a clinical version of the PFA generator, along with a circular catheter containing 9 electrodes, was utilized in an experiment involving 8 canines that were monitored for 12 weeks. The study conducted a comparative analysis between RFA and PFA procedures administered within pulmonary veins, and regular CT scans were performed to monitor the progression of stenosis. While severe stenosis and collateral injuries, including lung and esophageal damage as well as phrenic nerve impairment, were evident in cases involving RFA, no such adverse effects were observed in the group treated with PFA [53]. These consistent outcomes across various studies offer further reassurance regarding the safety profile of PFA in relation to the esophagus, strengthening its potential as a viable therapeutic option, which could minimize adverse effects and enhance patient outcomes.

## 5. Potential Drawbacks

In terms of safety, electroporation may face a potential concern known as arcing, wherein energy surpassing a certain threshold leads to the rapid accumulation of gas, generating a shock wave capable of producing barotrauma [59]. The arcing threshold can vary depending on the waveform and catheter design, implying the need to carefully evaluate individual PFA parameter settings. A recent study by Hsu *et al*. [50] reported no evidence of charring on the catheter tip. Likewise, there were no instances of steam pops, pericardial effusion, cardiac tamponade, or mural thrombus during the procedure, as confirmed by ICE and gross pathology examination. Additionally, no significant mechanical injury was observed on gross pathology, and there were no significant thromboembolic incidents detected in organs upstream, downstream, or within the heart [50].

Since PFA frequently leads to the formation of micro-bubbles, likely resulting from electrolysis, there is a valid concern about potential silent cerebral infarctions (SCI) associated with these micro-bubbles. However, reassuringly, a study conducted using canine models did not reveal any occurrence of SCI following the administration of PFA in the ascending aorta [60]. Furthermore, in the IMPULSE/PEFCAT trial, Reddy *et al*. [48] conducted cerebral magnetic resonance imaging (MRI) on 13 patients who underwent PFA, but no instances of SCI were reported. Nonetheless, when the same group used a focal PFA catheter, postprocedural brain MRI showed the presence of asymptomatic lesions in 9.8% of patients on diffusion-weighted imaging (DWI) or fluid-attenuated inversion recovery (FLAIR), while 5.9% of patients exhibited such lesions on DWI and FLAIR combined [61]. It is important to highlight that, in the context of CA for AF, the occurrence of SCI, which pertains to the presence of asymptomatic cerebral lesions identified through imaging modalities such as MRI, has been documented in as much as 67% of patients [62]. Further studies are required to clarify or rule out the occurrence of this phenomenon with currently available PFA catheters and, if present, assess potential ways to prevent it.

## 6. Currently Available Clinical Data

### 6.1 First Experience

In 2018, Reddy *et al*. [63] published the first-in-human experience using monophasic PFA during the ablation of paroxysmal AF. The group successfully performed PFA ablation in 22 patients at two centers, with excellent outcomes for catheter-based PVI and epicardial ablation. The procedure was reported as rapid and safe, demonstrating the potential for tissue-specific, ultrafast AF ablation. A Farawave (Farawave, Farapulse Inc, Menlo Park, CA, USA; formerly Iowa Approach) 12F over-the-wire catheter was used. Its distal portion consists of 5 splines, each featuring 4 electrodes per spline, with the third one capable of recording electrograms (Fig. 4A). This catheter must be deployed in a closed-basket configuration for gentle manipulation within the left atrium and is steered using a dedicated 13F sheath. Subsequently, it can be expanded into the flower configuration with a diameter of up to 31 mm. The study reported acute isolation of the PV in 100% of the cases, employing an average of 12.4 ± 1.0 lesions per patient (3.26 ± 0.5 lesions per pulmonary vein). The administered voltages spanned from 900 V to 2500 V, leading to an average delivery of 78 J per procedure. The left atrial dwell time was approximately 26 ± 4.3 minutes. As a result, the study demonstrated a high success rate and significantly reduced procedural times [63].

### 6.2 Subsequent Clinical Trials

Reddy *et al*. [48] later conducted two trials, IMPULSE, and PEFCAT, including patients with symptomatic paroxysmal AF who underwent PVI using the Farapulse system. In the IMPULSE trial (n = 40), monophasic waveforms with voltages between 900 and 1000 V were utilized under general anesthesia and with paralytic agents. In the PEFCAT trial (n = 41), biphasic waveforms with voltages between 1800 and 2000 V were employed, and most patients underwent the procedure under conscious sedation, which was well-tolerated despite some patients experiencing cough during the PFA delivery. Notably, there were no reconnections despite adenosine testing 20 minutes after the last PFA application. While phrenic nerve capture was observed while applying PFA to the right PV, none of the patients experienced phrenic nerve palsy. Notably, in the PEFCAT trial, adjustments to the biphasic waveform led to a substantial increase in the achievement of durable electrical isolation, rising from 63% to 100% during repeat electroanatomic mapping conducted at a median of 84 days following the initial ablation procedure. In both trials, PVI was successfully achieved in all patients, encompassing 81 individuals. This was accomplished with an average of 6.4 ± 2.3 applications and an energy delivery of 78 J per PV. Overall, these studies underscore promising outcomes associated with PFA with the Farapulse system for the management of AF [48].

A comparative analysis was conducted between two patient groups: one consisting of 37 individuals who underwent post-procedural CT reconstruction of the LA 3 months following PFA ablation as part of the IMPULSE and PEFCAT trials, and another comprising 43 control patients who had received RFA pulmonary vein isolation (PVI) in the TOCCASTAR and HEARTLIGHT trials. The results demonstrated that within the PFA cohort, only 0.8% of the PVs exhibited mild stenosis, characterized by a 30–49% reduction in either the long or short axis. In contrast, the RFA cohort displayed 11.4% with mild stenosis, 1.8% with moderate stenosis (50–69% reduction), and 1.2% with severe stenosis (70–100% reduction) of the PVs (*p* < 0.001). These findings indicate that PFA is linked to a substantially lower risk of pulmonary vein stenosis in comparison to RFA [64].

Loh *et al*. [65] shared their experience using a variable loop (16–27 mm) 14-polar catheter for PFA. They delivered PFA using 200 J monophasic pulses with an external defibrillator to achieve PVI in 10 patients, successfully isolating all 40 pulmonary veins. However, during the procedure, 9 out of the 10 patients exhibited transient ST segment elevation in the inferior leads. Although the exact cause of this elevated ST segment is unclear, the authors hypothesize that it is unlikely due to ischemia, as the changes appeared immediately after energy delivery, unlike typical ischemia-related ST segment changes. Instead, the authors believe that the electrical depolarization induced by the energy field might be responsible for these transient ST segment changes. It’s worth noting that this high prevalence of ST segment changes was not observed in other studies, raising the possibility that the specific type of energy used (monophasic 200 J shocks) could be the contributing factor. As a result, the safety of this approach requires further investigation and evaluation [65].

Similarly, Duytschaever *et al*. [66] recently published 1-year-follow-up results from the inspIRE study, which aimed to assess the safety and effectiveness of a fully integrated biphasic PFA system with a variable-loop circular catheter (Fig. 4C) for treating paroxysmal AF. PVI was performed using the Varipulse PFA system, with at least 12 applications per vein and confirmation of entrance block with adenosine/isoproterenol. The study involved two waves: Wave I for initial safety assessment and Wave II for pivotal phase testing. Across 13 centers in Europe/Canada, 226 subjects underwent PFA. The study confirmed the safety of this PFA system, as no esophageal thermal lesions or pulmonary vein stenosis were observed. Enhancements in workflow resulted in a reduction of silent cerebral lesions. In Wave II, no primary adverse events were reported. With a 100% entrance block, 97.1% of targeted veins achieved PVI without acute reconnection. The study demonstrated a high rate of freedom from symptomatic atrial arrhythmia recurrence at 12 months (78.9%) and a low repeat ablation rate (7.7%). The inspIRE trial confirmed the safety and effectiveness of another novel mapping integrated PFA system [66].

More recently, Reddy *et al*. [67] published a study investigating the application of PFA through the Farapulse system in patients with persistent AF. They conducted a single-arm study involving 25 patients, wherein they performed PVI, posterior wall isolation (PWI), and CTI isolation procedures. Successful PVI was accomplished using a Farawave catheter or a standard multielectrode mapping catheter to construct a voltage map of the LA. Subsequently, a pentaspline catheter featuring 4 electrodes in each spline was employed for PWI in 24 patients, applying PFA with biphasic waveforms ranging from 1600 to 2000 V per application, with a median PFA duration of 22 minutes. CTI lines were created using a focal PFA catheter in 13 patients, achieving acute bidirectional block in all instances. Following a remapping procedure 2.5 months later, PVI, PWI, and CTI lesions remained isolated in 96%, 100%, and 100% of cases, respectively [67]. Likewise, Verma *et al*. [68] recently published findings from the PULSED AF pivotal study. This was a prospective, global, multicenter, nonrandomized, paired single-arm investigation in patients who underwent PFA with paroxysmal (n = 150) or persistent (n = 150) symptomatic AF with the PulseSelect PFA System. The study showcased the efficacy of PFA, with a success rate of 66.2% for patients with paroxysmal AF and 55.1% for those with persistent AF at the 1-year follow-up mark. It’s worth highlighting that the primary safety endpoint was achieved in merely 0.7% of patients in both the paroxysmal and persistent AF groups [68]. These consistent outcomes across various systems indicate that PFA might represent a secure and efficient approach to perform PVI, PWI, and CTI line in cases of persistent AF, demonstrating highly favorable results. However, 1-year freedom from AF is, at best, comparable and perhaps modestly inferior to results from modern RF or cryoablation studies [8].

### 6.3 Prospective Registries

In 2021, Reddy *et al*. [69] published 1-year outcomes for 3 multicenter trials evaluating the success of PFA of AF. The trials included were IMPULSE, PEFCAT, and PEFCAT II. A total of 121 patients with paroxysmal AF underwent PVI using the Farapulse system. Acute PVI was achieved in 100% of the pulmonary veins with PFA alone. Invasive remapping performed approximately 2 to 3 months after the initial ablation demonstrated durable PVI in 84.8% of pulmonary veins (64.5% of patients) when using the optimized biphasic energy PFA waveform. Furthermore, 96.0% of the pulmonary veins (84.1% of patients) treated with the optimized PFA waveform maintained durable PVI. Regarding safety, primary adverse events occurred in only 2.5% of patients, including 2 cases of pericardial effusion or tamponade and 1 hematoma. Additionally, there was 1 transient ischemic attack observed. The 1-year Kaplan-Meier estimates for freedom from any atrial arrhythmia were 78.5 ± 3.8% for the entire cohort and 84.5 ± 5.4% for the group treated with the optimized biphasic energy PFA waveform. Overall, the results demonstrate that PVI using a “single-shot” PFA catheter offers excellent PVI durability and acceptable safety, with a low 1-year rate of atrial arrhythmia recurrence. The data support the efficacy of the nonthermal ablative mechanism of PFA in achieving clinical success without compromising safety. These findings have significant implications for the clinical application of PFA in patients with paroxysmal AF, suggesting it is a promising ablation strategy for PVI with favorable outcomes at 1 year [69].

In July 2023, Schmidt *et al*. [70] published results from the EUropean Real World Outcomes with Pulsed Field AblatiOn in Patients with Symptomatic AtRIAl Fibrillation (EU-PORIA) registry. This registry aims to assess the safety, efficacy, and learning curve characteristics of PFA using the Farapulse system for PVI in patients with AF. The registry included 1233 AF patients from 7 high-volume centers, treated by 42 operators. The procedure achieved a high success rate with a low major complication rate (1.7%), including pericardial tamponade and transient ischemic attack or stroke. The Kaplan-Meier estimate of arrhythmia-free survival at 1-year follow-up was 74%, with no significant influence from operator experience [70]. Similarly, Turagam *et al*. [71] recently reported results from the MANIFEST-PF multinational retrospective patient-level registry investigating the safety and effectiveness of PFA. The registry included 1568 patients with AF who underwent PFA at 24 European centers. PVI was achieved in 99.2% of patients, and the 1-year Kaplan-Meier estimate for freedom from atrial arrhythmia was 78.1%, with better outcomes in patients with paroxysmal AF compared to persistent AF. Acute major adverse events occurred in 1.9% of patients [71]. The ADVANTAGE-AF (NCT05443594) and AdmIRE (NCT05293639) trials are currently running single-arm studies that will provide further data about long-term clinical outcomes associated with PFA using the Farapuse and Varipulse PFA systems, respectively. Through these observational multicenter studies, PFA demonstrated favorable results, providing an excellent safety profile and short procedure times in a real-world AF patient population.

## 7. Catheter Ablation with PFA vs. RFA and Cryoablation

Although promising medium-term results have been confirmed by repeated trials and registries, studies regarding a direct comparison with well-established energy sources for CA of AF are needed. Providing data in this regard, Urbanek *et al*. [72] performed a retrospective study to compare the procedural and long-term outcomes of cryoablation vs. PFA in patients with AF. Four hundred patients were included, with 200 undergoing cryoballoon (CB)-based PVI and 200 undergoing PFA using a pentaspline catheter. Acute PVI success was achieved in 100% of PFA and 98% of CB patients. The median procedure time was significantly shorter for PFA compared to CB. Overall procedural complications were higher in CB, mainly driven by a higher rate of phrenic nerve palsies. However, the 1-year success rates for both paroxysmal and persistent AF were similar for both techniques [72]. On the other hand, Badertscher *et al*. [73] recently carried out a nonrandomized prospective study comparing high-power, short-duration RFA with PFA for the ablation of AF. Comparable to the results reported by Urbanek *et al*. [72], PFA was associated with shorter procedural time with similar freedom of AF at 6 months of follow-up [73]. Albeit these studies were the first reported attempts at comparing PFA with other ablation energy sources, randomized trials are essential to objectively compare outcomes.

Reddy *et al*. [74] recently addressed this gap with the ADVENT trial. This is a multicenter, prospective, single-blind, randomized controlled study that compares the safety and efficacy of PFA using a multielectrode pentaspline catheter with conventional thermal ablation (either cryoballoon or radiofrequency ablation) for treating drug-resistant paroxysmal AF. The trial’s primary effectiveness endpoint includes acute procedural success and freedom from any documented atrial arrhythmia recurrence, repeat ablation, or use of antiarrhythmic drugs after a 3-month post-ablation blanking period. The primary safety endpoint comprises acute and chronic device- and procedure-related serious adverse events. The study reported that the PFA catheter is non-inferior to standard-of-care thermal ablation in freedom from the primary composite endpoint. Similarly, the study reported no difference in acute and chronic device- and procedure-related complications [75]. Nevertheless, is it worth noting that a larger sample may be required to assess for differences in complication given the low rates of complications associated with current RFA power delivery and ventilation strategies [34, 76].

## 8. Conclusion 

The comprehensive analysis of the literature highlights the significant potential of PFA as a revolutionary nonthermal ablation modality for treating AF. PFA offers an innovative approach by utilizing rapid electrical pulses to induce IRE, enabling precise myocardial tissue ablation while sparing collateral cardiac structures from thermal injury. The findings from IMPULSE, PEFCAT, PEFCAT II, and inspIRE trials underscore the safety and efficacy of PFA in achieving durable PVI and reducing the risk of thermally mediated complications. The MANIFEST-PF and EU-PORIA registries further strengthen the evidence, demonstrating favorable clinical outcomes and minimal adverse events associated with PFA in real-world scenarios. Moreover, direct comparisons with conventional thermal ablation techniques reveal that PFA shows comparable procedural effectiveness and shorter procedure times, with no occurrences of phrenic nerve palsies. While these results are promising, further research and ongoing clinical trials are imperative to validate the long-term success and safety of PFA compared to existing ablation modalities. As PFA continues to evolve, it holds the potential to revolutionize AF ablation procedures, providing patients and clinicians with a safe and effective alternative to conventional thermal ablation methods.

## Abbreviations

AF, atrial fibrillation; ATP, adenosine triphosphate; CA, catheter ablation; CB, cryoballoon; CT, computed tomography; CTI, cavotricuspid isthmus isolation; DC, direct current; DWI, diffusion-weighted imaging; FLAIR, fluid-attenuated inversion recovery; ICE, intracardiac echocardiography; IRE, irreversible electroporation; MRI, magnetic resonance imaging; PFA, pulsed-field ablation; PV, pulmonary vein; PVI, pulmonary vein isolation; PWI, posterior wall isolation; RCT, randomized control trial; RF, radiofrequency; RFA, radiofrequency ablation; SCI, silent cerebral ischemia.
